# Exploring advanced nanostructures and functional materials for efficient hydrogen storage: a theoretical investigation on mechanisms, adsorption process, and future directions

**DOI:** 10.3389/fchem.2025.1525140

**Published:** 2025-02-11

**Authors:** Gourhari Jana, Pratim Kumar Chattaraj

**Affiliations:** ^1^ School of Chemical Sciences, Indian Association for the Cultivation of Science, Kolkata, India; ^2^ Department of Chemistry, Birla Institute of Technology, Ranchi, Jharkhand, India

**Keywords:** promising H_2_ storage materials, energy storage, hydrogen storage mechanisms, nanostructures, mechanistic advancements

## Abstract

Hydrogen is a promising candidate for renewable energy storage and transportation due to its high energy density and zero carbon emissions. Its practical applications face challenges related to safe, efficient storage and release systems. This review article investigates advanced nanostructured materials for hydrogen storage, including metal acetylide and cyanide complexes, B,N-doped γ-graphyne nanotubes (γ-GNT), lithium-phosphide double helices, and Ni-decorated carbon-based clusters. Density Functional Theory (DFT) based computations are used to analyze binding energies, thermodynamic stability, and adsorption mechanisms. Ni-decorated C_12_N_12_ nanoclusters demonstrate enhanced storage capacities, binding up to eight H_2_ molecules with a favorable N-(μ-Ni)-N configuration. Lithium-phosphide double helices show potential for 9.6 wt% hydrogen storage within a stable, semiconducting framework. Functionalization of γ-GNT with OLi_2_ at boron-doped sites significantly enhances storage potential, achieving optimal hydrogen binding energies for practical applications. Additionally, metal acetylide and cyanide complexes, stabilized by noble gas insertion, display thermodynamically favorable hydrogen adsorption. These results highlight the potential of these functionalized nanostructures for achieving high-capacity, reversible hydrogen storage. The nanostructures in this study, such as γ-graphyne nanotubes (γ-GNT), lithium-phosphide double helices, metal acetylide and cyanide complexes, and Ni-decorated carbon-based clusters, are selected based on their ability to exhibit complementary hydrogen adsorption mechanisms, including physisorption and chemisorption. γ-GNT offers high surface area and tunable electronic properties, ideal for physisorption enhanced by heteroatom doping. Lithium-phosphide double helices facilitate Kubas-like chemisorption through unsaturated lithium centers. Metal acetylide and cyanide complexes stabilize hydrogen adsorption via charge transfer and conjugated frameworks, while Ni-decorated clusters combine polarization-induced physisorption. These materials represent a strategic approach to addressing the challenges of hydrogen storage through diverse and synergistic mechanisms. The review also addresses challenges and outlines future directions to advance hydrogen’s role as a sustainable fuel.

## 1 Introduction

As the world continues to confront the challenges posed by climate change, the search for clean and sustainable energy sources has become a critical priority. Hydrogen storage is one of the most critical challenges in achieving a hydrogen-based energy economy. Among the many alternatives to fossil fuels, hydrogen stands out as a promising candidate due to its high energy density, zero carbon emissions, and potential for use across various industries, including power generation, transportation, and industrial applications. When used as a fuel, hydrogen only produces water vapor as a byproduct, making it an environmentally friendly alternative to conventional energy sources like coal, oil, and natural gas. Furthermore, hydrogen’s versatility as an energy carrier makes it an attractive solution for reducing greenhouse gas emissions and dependence on fossil fuels. Despite these advantages, the large-scale adoption of hydrogen as a mainstream energy source faces significant barriers, particularly related to the efficient storage and safe release of hydrogen. To overcome these challenges, significant research efforts are being directed toward the development of advanced materials and methods for hydrogen storage. The hydrogen molecule (H_2_), while energy-dense, is small and lightweight, presenting inherent difficulties in achieving high volumetric and gravimetric storage densities in practical settings. Various theoretical and computational studies have provided insights into the chemical interactions and thermodynamics underlying hydrogen storage and release, which are fundamental to the development of effective storage materials (Varin et al., 2009; Staubitz et al., 2010; Bluhm et al., 2006; Furukawa et al., 2013; Srinivasan and Sankaranarayanan, 2017; Murdock et al., 2017; Gupta and Liu, 2015; Orimo and Fujii, 2000; Carter, 2011).

Quantum mechanical methods, such as DFT, are extensively used to study hydrogen binding energies and adsorption mechanisms. These methods also provide insights into the stability of potential storage materials at the atomic level. Theoretical insights are essential for predicting material behaviors. They enable the targeted design and optimization of materials with desirable hydrogen storage properties (Powell et al., 2009; Hirscher and Yartys, 2010; Schlapbach and Züttel, 2001). The storage challenge encompasses two primary approaches: (1) physical storage, which relies on compression or liquefaction of hydrogen gas, and (2) chemical storage, which involves the absorption or adsorption of hydrogen within materials. Physical storage methods often require high pressures (up to 700 bar) or cryogenic temperatures to achieve viable hydrogen densities, both of which are energy-intensive and introduce safety concerns. Therefore, recent research has emphasized chemical storage approaches, where hydrogen is stored within materials through physisorption, chemisorption, or covalent bonding, offering potentially safer and more energy-efficient alternatives. Metal hydrides are among the most extensively studied hydrogen storage materials due to their high volumetric hydrogen densities.

Over the past decade, research used on the development of advanced nanostructured materials to address these challenges, especially carbon-based materials due to their tunable surface chemistry, high surface area, and potential for lightweight storage solutions. The development of advanced materials capable of addressing this challenge requires a fundamental understanding of their interaction mechanisms with H_2_ molecules. We have selected metal acetylide complexes, γ-graphyne nanotubes (γ-GNT), lithium-phosphide helices, and Ni-decorated clusters as representative systems based on their distinct structural, electronic, and adsorption properties that align with the desired characteristics for hydrogen storage: high capacity, reversibility, and stability. Metal acetylide complexes are chosen due to their ability to stabilize metal centers while allowing for strong yet reversible interactions with hydrogen molecules through Kubas-type interactions. Studies by Rosi et al. (2003), Wang and Yan (2016) and Latroche et al. (2006), Heinekey and Oldham (1999) have emphasized the importance of these interactions in achieving efficient storage. Similarly, γ-GNTs have been explored for their unique sp-hybridized carbon framework, offering a combination of high surface area and tunable electronic properties conducive to hydrogen adsorption (Berber et al., 2000; Liu and Cui, 2014. Lithium-phosphide helices stand out for their novel double-helix structure and high hydrogen storage capacities, which are facilitated by their lightweight nature and strong chemisorption capabilities. Previous computational studies (Kim et al., 2017; Bhatia et al., 2019; Hanada et al., 2004; Crabtree, 2005) have shown their potential for achieving high gravimetric densities, critical for practical applications. Lastly, Ni-decorated clusters have been extensively investigated for their high adsorption energies and ability to polarize H_2_ molecules, as highlighted by Wang et al. (2015), Latroche (2010) and Zhang et al. (2018), Van Bavel and Yang (2017). Seminal studies by Zhou et al. (2008) and Hirscher et al. (2010), along with contributions from other leading researchers, offer valuable insights into the mechanisms of hydrogen adsorption and the design of advanced storage materials. Advanced hydrogen storage materials, particularly those based on metal-organic frameworks (MOFs), nanostructured carbons, and other nanomaterials, have garnered significant attention due to their potential in meeting the hydrogen storage demands for sustainable energy solutions. The hydrogen adsorption mechanisms, including physisorption and chemisorption, are pivotal in designing materials that exhibit both high capacity and reversibility. Aromatic clusters, as discussed by Pal and Chattaraj (2021), have emerged as promising materials for hydrogen storage due to their ability to provide favorable adsorption sites via their delocalized π-electron systems, which enhance interactions with hydrogen molecules. This capability is further supported by other studies that highlight the importance of electronic structure modifications, such as heteroatom doping, in improving the storage capacity and stability of hydrogen adsorption (Wang et al., 2018a; Zhang et al., 2020; Li Y. et al., 2021; Wang X. et al., 2018; Zhou Y. et al., 2019; Dey et al., 2018; Yang et al., 2020; Ghosh et al., 2021; Zhang et al., 2021; Wang et al., 2020; Zhang et al., 2019; Liu et al., 2020; Zhao L. et al., 2020; Yadav et al., 2021; Jiao et al., 2021; Ghosh and Saha, 2020; He et al., 2019; Zhou L. et al., 2019; Zhang et al., 2020; Xie et al., 2020). These advancements underscore the potential of aromatic clusters and similar materials for developing high-performance hydrogen storage systems. Additionally, computational approaches such as density functional theory (DFT) have been instrumental in understanding and predicting the hydrogen binding energies and structural stabilities of these materials (Wang et al., 2018b; Zhang et al., 2021; Li Y. et al., 2021; Wang X. et al., 2018). Researchers have also explored the role of transition metal dopants and the functionalization of nanostructured materials to further enhance hydrogen uptake and retention properties (Dey et al., 2018; Yang et al., 2020). These studies underline the importance of tailoring the electronic and structural properties of materials to optimize their hydrogen storage performance and facilitate the transition towards clean energy systems. These materials collectively represent a spectrum of innovative approaches in the hydrogen storage domain Li Z. et al., 2021; Mo et al., 2018; Cychosz and Matzger, 2009, Rowsell and Yaghi, 2005, Wang et al., 2019. The doping of these structures with metals or polar functional groups has shown substantial promise in enhancing their hydrogen storage capacity by introducing specific binding sites for H_2_ molecules, thereby achieving favorable adsorption energies and structural stability.

Functionalization with metals, such as nickel, has been particularly effective in enhancing hydrogen binding energies. For instance, Ni-functionalized C_12_N_12_ nanoclusters demonstrate multiple adsorption configurations for H_2_, with binding energies ranging from 11.9 to 23.3 kcal/mol. This range is ideal for achieving reversible hydrogen storage, balancing strong adsorption for stability and moderate desorption energies for practical release. The strategic placement of Ni atoms on C_12_N_12_ helps create optimal sites for hydrogen storage. This allows the uptake of up to eight H_2_ molecules per cluster. It highlights the importance of electrostatic and orbital interactions in stabilizing hydrogen on metal-decorated nanostructures. Energy decomposition analysis (EDA) has shown that polar Ni-C bonds play a crucial role in the adsorption of H_2_ molecules. Charge transfer interactions significantly stabilize these adsorptions, highlighting the adaptability and efficiency of metal-functionalized carbon nanostructures for hydrogen storage applications (Jana et al., 2020).

Beyond metal-functionalized systems, lithium phosphide (Li_n_P_n_) double helices have emerged as potential hydrogen storage materials due to their unique helical structures, which allow for efficient hydrogen adsorption. Studies have shown that each lithium center within the Li_n_P_n_ structure can bind up to two H_2_ molecules, yielding a hydrogen uptake capacity of up to 9.6 wt%—a promising result for practical hydrogen storage applications. The binding energies per H_2_ molecule on helical structures range between 1.7 and 3.2 kcal/mol, which is optimal for maintaining hydrogen stability under ambient conditions while facilitating controlled release. This balance makes these materials suitable for on-demand hydrogen storage applications, highlighting their practical potential in energy systems (Jana et al., 2018a).

An innovative strategy focuses on functionalizing γ-GNTs with polar molecules like OLi_2_. These molecules are precisely anchored to boron-doped hexagonal (h-BN) sites, leveraging their localized electronic properties to enhance hydrogen adsorption efficiency. This functionalization method optimizes the interaction between the polar molecules and the substrate, creating highly favorable conditions for hydrogen storage. DFT calculations reveal that the functionalization of γ-GNTs with OLi_2_ not only stabilizes the structure but also significantly enhances hydrogen adsorption capacity due to the strong dipole field created by the OLi_2_ group. Each OLi_2_ group on γ-GNT can adsorb up to eight H_2_ molecules, with binding energies between 0.2 and 0.6 eV, which is an ideal range for reversible practical conditions. The distinctive electronic structure of OLi_2_-functionalized γ-GNT, as highlighted by natural bond orbital (NBO) analysis, exhibits a pronounced charge separation between the lithium and oxygen atoms. This polarization facilitates the effective adsorption of H_2_ molecules, with the positively charged lithium atoms and negatively charged oxygen atoms serving as complementary binding sites. Such interactions enable high-capacity and reversible hydrogen storage, showcasing the potential of this functionalized nanostructure for advanced energy applications (Jana et al., 2018b).

Further, this review study includes the potential of metal acetylide and cyanide compounds, enhanced with noble gas atoms (Ng) and metals like Cu, Ag, and Au, for hydrogen storage applications. Through DFT calculations, we assessed adsorption energies, Gibbs free energies, and charge distributions on atomic centers, complemented by energy decomposition analysis, to explore configurations in which up to three hydrogen molecules are adsorbed on the metal center. Our results indicate that both MNgCCH and MNgCN compounds can effectively accommodate up to three hydrogen molecules, adopting distinct geometric configurations: single hydrogen adsorption favors a “T-shaped” structure, double hydrogen adsorption forms a “Y-shaped” configuration, and triple hydrogen systems adopt a “Td-like” geometry. The negative values of Gibbs free energy confirm that hydrogen adsorption occurs spontaneously, highlighting the compounds’ promise as efficient, reversible hydrogen storage materials. These insights offer a valuable framework for designing next-generation materials with high hydrogen storage capacity (Jana et al., 2021).

This review summarizes the latest advancements in hydrogen storage materials, specifically focusing on carbon-based nanostructures and metal-functionalized complexes. We have carried out computational analyses to reveal the mechanisms of hydrogen adsorption. Additionally, we have investigated the structural stability and electronic characteristics of these materials. By addressing the limitations and opportunities in the field, we aim to outline a pathway for the design of next-generation materials capable of meeting the demands of sustainable hydrogen energy storage.

## 2 Theoretical background and computational modeling

In order to understand and develop materials for hydrogen storage, theoretical and computational chemistry are crucial because they enable researchers to predict material properties, investigate atomic-scale mechanisms, and optimize hydrogen storage performance without requiring a great deal of trial-and-error experimentation.

Among several most popular computational methods in the investigation of hydrogen storage research, DFT is one of the reliable and widely used methods. From electronic structures and reaction mechanisms to adsorption behaviors and thermodynamic features, each technique offers a distinct perspective on various facets of hydrogen storage.

To identify and design more efficient hydrogen storage materials, it is essential to understand the fundamental chemical interactions between hydrogen and the storage material. This understanding requires in-depth knowledge of the material’s electronic structure, thermodynamic properties, and the nature of the hydrogen-material interaction. Quantum mechanical methods, such as DFT, have proven to be invaluable tools for investigating these interactions at the atomic level.

Theoretical studies using DFT allow researchers to predict the stability of hydrogen adsorbed systems, the adsorption energies, and the equilibrium conditions for hydrogen storage and release. DFT simulations provide insight into the nature of hydrogen bonding with the material, whether through physisorption (weak van der Waals forces), chemisorption (stronger covalent bonding), or a combination of both. By examining the adsorption energies and the changes in free energy upon hydrogen adsorption and desorption, researchers can assess the feasibility of a material for practical hydrogen storage applications.

In particular, DFT calculations help in identifying optimal storage materials by revealing key factors that influence hydrogen storage, such as the strength of the interaction between hydrogen and the material, the material’s ability to undergo reversible hydrogenation and dehydrogenation cycles, and the overall energy efficiency of the process. By focusing on the thermodynamics of hydrogen adsorption, these theoretical insights can guide the design and optimization of novel materials that meet the stringent requirements for practical hydrogen storage systems. We have employed DFT-based methods in predicting the electronic factors controlling hydrogen adsorption energy. We recognize the critical importance of providing a comprehensive discussion on this aspect to enhance the reliability and robustness of the findings. A thorough comparative analysis of the DFT methods are used, such as the Generalized Gradient Approximation (GGA), meta-GGA, and hybrid functionals, alongside the effect of different basis sets on the calculated adsorption properties. Specifically, we have explored the performance of functionals like PBE, B3LYP, and M06-L, which have been commonly employed in similar studies (Bai et al., 2019; Li J. et al., 2020). This analysis highlights the varying accuracy of these functionals in predicting the binding energies and adsorption mechanisms, thus ensuring that the results are robust and reliable. Furthermore, the DFT-calculated adsorption properties with data from wave function-based methods, such as coupled-cluster theory or Møller–Plesset perturbation theory is dicussed by Barone et al. (2021), to validate and contextualize our findings and the concern about methodological rigor in predicting hydrogen storage properties. A comprehensive comparative discussion on how different DFT methods influence the calculated adsorption energies and electronic properties of materials would indeed strengthen the quantum chemical analysis presented in this study. DFT methods, including generalized gradient approximation (GGA), meta-GGA, and hybrid functionals, are essential tools for evaluating hydrogen adsorption mechanisms. However, the choice of the functional significantly impacts the predicted binding energies and adsorption behaviors due to the inherent differences in how these functionals treat electron correlation effects. For instance, GGA functionals, such as PBE, often underestimate the adsorption energies because they fail to accurately capture long-range van der Waals interactions. This leads to less reliable predictions for weak physisorption systems, like hydrogen adsorption on porous materials Cohen et al. (2021). Meta-GGA functionals, such as the TPSS functional, improve upon GGA by better describing exchange-correlation interactions, particularly in systems with significant electron correlation effects Zhao X. et al. (2020). Hybrid functionals, such as B3LYP, combine the advantages of both local and nonlocal exchange-correlation effects, often yielding more accurate adsorption energies and electronic properties, particularly for systems where charge transfer and orbital overlap play significant roles (Natarajan et al., 2022). These differences in functionals directly influence the calculated hydrogen adsorption energies, making it crucial to consider multiple methods for reliable predictions.

Additionally, structural alterations such as the introduction of dopants or functional groups to a material can significantly affect both the electronic properties and hydrogen adsorption capabilities. For example, doping with electronegative atoms like nitrogen or oxygen can create localized charge densities that enhance the material’s ability to bind hydrogen via both physisorption and chemisorption mechanisms (Li et al., 2020b). In the case of graphene-based materials, the introduction of metal atoms or functional groups (e.g., carboxyl or hydroxyl groups) can modify the electronic density, alter the local electronic structure, and subsequently change the adsorption sites for hydrogen molecules, leading to enhanced adsorption capacities (Wang J. et al,. 2018).

## 3 Methodology and tools used

The geometry optimization and subsequent frequency analysis of all the systems are carried out at using DFT functionals and is reported to be suitable in interactions between molecular hydrogen and adsorption materials. To ensure a thorough understanding of the temperature- and pressure-dependent properties, an explicit description of the computational methodology used to calculate these factors is essential. In the context of hydrogen adsorption, temperature and pressure play a significant role in determining the spontaneity and feasibility of adsorption. One commonly employed approach for calculating temperature-dependent properties is the harmonic oscillator approximation (HOA), which assumes that the vibrational modes of the adsorbed hydrogen molecules can be treated as harmonic oscillators. This approximation allows for the calculation of partition functions, which are then used to estimate thermodynamic properties such as free energy, entropy, and enthalpy (Johnson et al., 2018).

To calculate pressure-dependent properties, the ideal gas law or a more sophisticated equation of state (e.g., van der Waals or virial equations) can be used. In many cases, the adsorption isotherms are constructed by varying pressure at a fixed temperature, allowing for the determination of adsorption capacities and their dependence on pressure. For accurate modeling, one must consider a range of pressures typically encountered in practical storage applications, such as those under ambient conditions or high-pressure environments (Zhao et al., 2021).

For instance, in studies investigating hydrogen storage materials, the temperature range often spans from 77 K (liquid nitrogen temperature) to room temperature (298 K) to simulate cryogenic and ambient storage conditions. The pressure range typically considers both low-pressure (up to 1 atm) and high-pressure scenarios (up to 100 MPa) to assess the storage potential under various operational conditions (Li et al., 2020c).

Incorporating these considerations into the study of hydrogen adsorption helps determine the feasibility of the material for real-world applications. Specifically, the temperature and pressure dependence of the adsorption process allows researchers to assess whether the adsorption is spontaneous (negative Gibbs free energy) and reversible, which are critical factors for practical hydrogen storage systems. The detailed explanation of these methods provides clarity and ensures that the calculated adsorption properties align with experimental observations and real-world conditions.

To calculate average adsorption energies (*E*_ads_), and free energy change (∆*G*_ads_) per H_2_ molecule the following expressions have been used:

$$E_{ads}=1/n\;\left\{E_{nH2\;\cdot \cdot \cdot \;\text{systems}}-\left(E_{systems}+nE_{H2}\;\right)\right\}$$

$$\Delta G_{ads}=1/n\;\left\{G_{nH2\;\cdot \cdot \cdot \;\text{systems}}-\left(G_{systems}+nG_{H2}\;\right)\right\}$$

All these computations are done using Gaussian 09 program package. The natural population analysis (NPA) scheme is adopted to compute atomic charges (QNPA).

To ensure a robust and comprehensive understanding of the computational modeling employed in this study, a detailed description of the periodic Density Functional Theory (DFT) calculations is provided. The computational setup includes the choice of supercell, k-point sampling, and relevant parameters such as convergence criteria, pseudopotentials, and exchange-correlation functionals, all of which are carefully selected to provide accurate results for the hydrogen adsorption study. The supercell selection plays a crucial role in periodic DFT calculations, as it defines the repeating unit that models the material. A sufficiently large supercell is chosen to minimize interactions between periodic images and ensure that the system behaves as close to an infinite material as possible. The k-point sampling, which discretizes the Brillouin zone for numerical integration, is optimized to balance computational efficiency with accuracy. Typically, a denser k-point grid is required for more accurate results, especially for materials with low symmetry or complex electronic structures (Kresse and Furthmüller, 1996; Perdew et al., 1996).

In periodic DFT calculations, the choice of the exchange-correlation functional is critical for accurate prediction of adsorption energies. For example, the generalized gradient approximation (GGA), hybrid functionals, or meta-GGA functionals are used depending on the system’s complexity and the need for capturing dispersion interactions (Grimme et al., 2010). The application of periodic boundary conditions ensures that the system’s behavior accurately reflects the extended nature of the material, which is particularly important for modeling materials like LiP helices, where long-range interactions between atoms play a significant role in determining hydrogen adsorption properties (Jain et al., 2013). In contrast, while cluster models are computationally less expensive and easier to apply, they introduce edge effects that can distort the material’s electronic structure. Cluster models are typically limited to a finite number of atoms and may fail to account for the long-range interactions present in periodic systems, thus underestimating or misrepresenting the energetics of hydrogen adsorption. Periodic models, by simulating infinite systems, more accurately reflect the material’s behavior in practical applications, such as hydrogen storage, by including the effects of the material’s extended electronic structure and periodicity (Todorov et al., 2007).

Thus, the advantages of periodic modeling are particularly evident in this study, as they allow for a more accurate description of the electronic structure and adsorption energetics, which are critical for understanding the performance of materials like LiP helices for hydrogen storage.

## 4 Results and discussion

In the quest for practical hydrogen storage materials, recent advancements have employed a combination of theoretical insights, computational studies, and experimental validation. Here, we summarize the key findings from computational studies and discuss how these results align with experimental observations, focusing on carbon-based nanostructures of Li_n_P_n_ double helix, B,N-doped nanotubes, Metal acetylides and cyanides.

There are some discussions on very recent developments in multiscale molecular systems for hydrogen storage.

### 4.1 Criteria for material selection

The nanostructures chosen for this study are selected based on their unique properties and the specific hydrogen adsorption mechanisms they enable. For instance, γ-graphyne nanotubes (γ-GNT) are known for their sp-sp^2^ hybridized carbon framework, which provides a high specific surface area, excellent chemical stability, and tunable electronic properties. These features make γ-GNT highly conducive to physisorption, as their extended *π*-electron cloud enhances weak van der Waals interactions with H_2_ molecules. Additionally, doping γ-GNT with heteroatoms (e.g., B or N) introduces localized charge density and improves binding through induced dipole interactions, effectively bridging the gap between physisorption and weak chemisorption.

In the case of lithium-phosphide double helices, their unique helical structure provides unsaturated lithium centers that act as open metal sites. These centers strongly favor chemisorption by facilitating charge transfer interactions. Specifically, H_2_ molecules interact via a Kubas-like mechanism, involving synergistic σ-donation from the H_2_ *σ*_g_ orbital to the metal center and *π*-back donation from the lithium d-orbital to the H_2_ *σ*_u*_ antibonding orbital. This interplay between physisorption at low temperatures and chemisorption under specific conditions offers exceptional hydrogen storage potential, as evidenced by their ability to reversibly adsorb and desorb H_2_.

For metal acetylide and cyanide complexes, the selection stems from their ability to stabilize hydrogen adsorption through highly polarizable metal centers and conjugated frameworks. The binding involves chemisorption driven by charge transfer from the H_2_ molecule to the metal center, while the conjugated ligands provide additional stabilization via resonance effects. These materials are particularly valuable for achieving strong binding energies under mild conditions, which are essential for practical hydrogen storage applications.

Ni-decorated carbon-based clusters, on the other hand, are selected for their proven ability to combine physisorption and chemisorption mechanisms effectively. Nickel atoms enhance hydrogen adsorption by providing localized unscreened charges and facilitating polarization-induced physisorption, while also enabling stronger chemisorption interactions through direct metal-H_2_ bonding.

This combination of physisorption, chemisorption, and structural diversity provides a comprehensive basis for our material selection. We believe these materials are not only representative of the state-of-the-art in hydrogen storage but also demonstrate complementary mechanisms that collectively address key challenges in this field.

#### 4.1.1 Hydrogen storage ability of carbon-based nanostructures: doping and mechanisms

**FIGURE 1:**
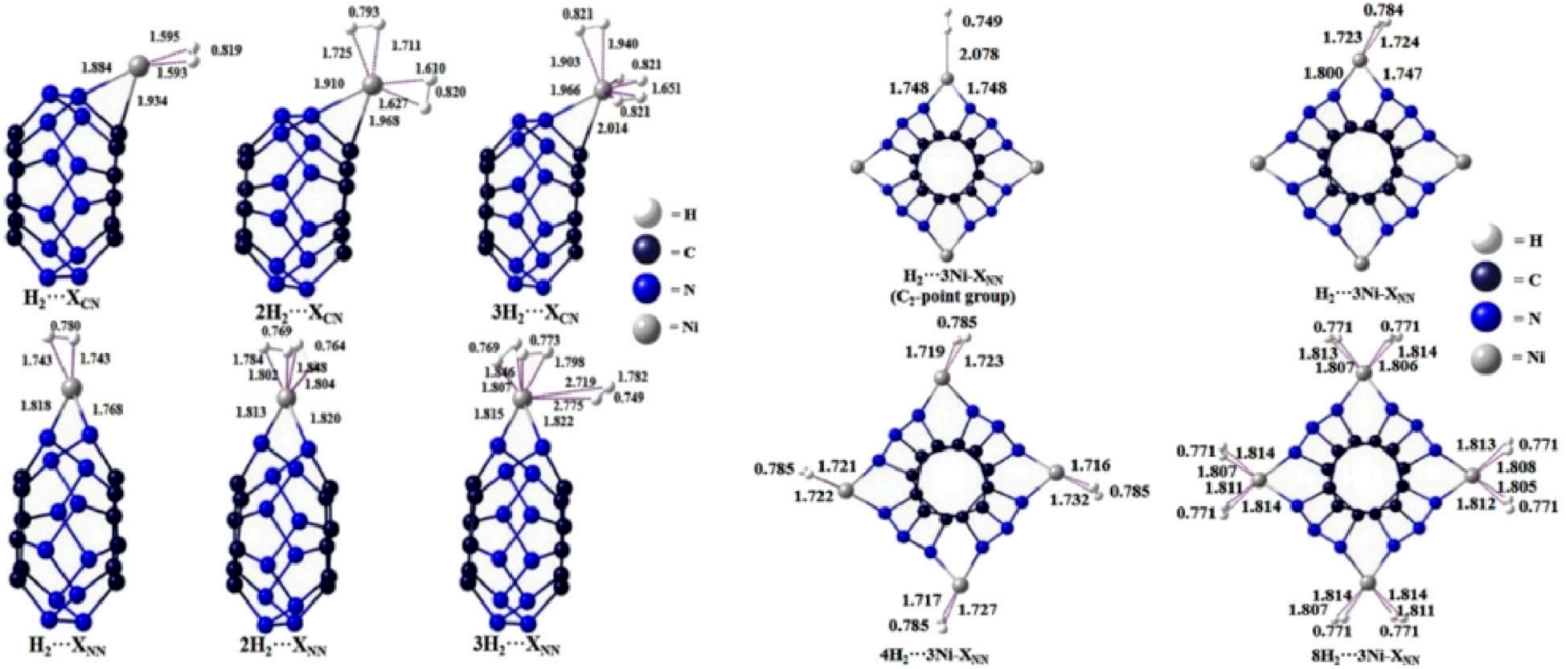
Optimized structures of H_2_ trapped single Ni bound C_12_N_12_ cluster at the ωB97X-D/Def2-TZVP level.

Our investigation into Ni-decorated C_12_N_12_ nanoclusters reveals promising insights into hydrogen storage capabilities, leveraging DFT calculations to explore various adsorption configurations. When Ni atoms are systematically anchored onto different bridging sites of the C_12_N_12_ framework, significant enhancements in hydrogen storage capacity are observed. Calculations of binding energies and free energies for Ni-C_12_N_12_ and H_2_…Ni-C_12_N_12_ configurations confirm the thermodynamic feasibility of hydrogen adsorption on these nanoclusters, suggesting their suitability as potential hydrogen storage materials (see Figure 1).

Natural Population Analysis (NPA) indicates that the polar Ni-C and Ni-N bonds drive the interaction with H_2_ molecules, promoting partial dissociation upon adsorption. The presence of a positively charged Ni atom on C_12_N_12_ allows for the adsorption of up to three H_2​_ molecules, with binding energies ranging from 11.9 to 23.3 kcal/mol in a C-(*μ*-Ni)-N bridging configuration, increasing to 16.0–39.2 kcal/mol for the N-(*μ*-Ni)-N arrangement. This indicates that the N-(*μ*-Ni)-N bridging mode stabilizes the adsorbed hydrogen more effectively, making it the preferred configuration for maximizing storage.

Extending this arrangement, the addition of four Ni atoms in an N,N-bridging mode yields a novel, thermodynamically stable structure with *D*_2d_ symmetry, optimized for hydrogen storage. Further analysis reveals that H_2_ molecules can adsorb in two configurations end-on and side-on a 4Ni-C_12_ N_12_ cluster, accommodating up to eight H_2_ molecules. This dual adsorption mode suggests a versatile and adaptable hydrogen storage system.

Additionally, EDA is performed considering H_2_ molecule(s) as one fragment and the Ni-C_12_N_12_ cluster as another (see Table 1). EDA highlights that the electrostatic and orbital interactions are the primary contributors to the interaction energy, the contribution from the former being 50.5–57.8% while that from the latter is 41.0–48.0% of the total attractive energy. On the other hand, the dispersion interaction is almost negligible (ca. 1.1–5.7% of the total attraction). Notably, the binding strength decreases slightly as additional H_2_ molecules are adsorbed, reflecting some degree of electron transfer from H_2_ to the Ni centers, further stabilizing the interactions. We have investigated the interaction energy (Δ*E*_int_) and the contribution of energy terms towards total interaction which are responsible for stabilizing the H_2_···Ni-C_12_N_12_ interaction.

**TABLE 1:** EDA results of H_2_ bound Ni-decorated Ni-C_12_N_12_ and 4Ni-C_12_N_12_ clusters at the revPBE-D3/TZ2P//ωB97X-D/Def2-TZVP. All energy values reported here are per H_2_ in kcal/mol. The values in parentheses are percentage contribution toward the total attraction, Δ*V*_elstat_ + Δ*E*_orb_ + Δ*E*_disp_.

Systems	Δ*E*_int_	Δ*E*_Pauli_	Δ*V*_elstat_	Δ*E*_orb_	Δ*E*_disp_
H_2_···Ni-C_12_N_12_(X_CN_)	−24.9	59.8	−48.9 (57.8)	−34.8 (41.0)	−1.0 (1.2)
2H_2_···Ni-C_12_N_12_(X_CN_)	−31.5	124.8	−86.2 (55.2)	−68.4 (43.7)	−1.8 (1.1)
3H_2_···Ni-C_12_N_12_(X_CN_)	−40.8	209.6	−126.5 (50.5)	−120.1 (48.0)	−3.8 (1.5)
H_2_···Ni-C_12_N_12_(X_NN_)	−10.7	49.3	−33.2 (55.4)	−25.4 (42.4)	−1.3 (2.2)
2H_2_···Ni-C_12_N_12_(X_NN_)	−15.3	83.5	−53.2 (53.9)	−42.8 (43.4)	−2.7 (2.7)
3H_2_···Ni-C_12_N_12_(X_NN_)	−18.0	88.7	−55.5 (51.9)	−45.2 (42.3)	−6.1 (5.7)
4H_2_···4Ni-C_12_N_12_(3Ni-X_NN_)	−44.9	213.0	−144.2 (55.9)	−108.5 (42.1)	−5.1 (2.0)
8H_2_···4Ni-C_12_N_12_(3Ni-X_NN_)	−60.3	328.0	−208.2 (53.9)	−169.1 (43.5)	−11.0 (2.8)

These findings emphasize the importance of strategic Ni placement to optimize adsorption properties and highlight the versatility of Ni-decorated C_12_ N_12_. as a promising material for next-generation hydrogen storage applications. Please see Figure 1 of H_2_ trapped single Ni bound C_12_N_12_ cluster.

#### 4.1.2 Hydrogen storage ability of Li_n_P_n_ double helix

Hydrogen is highly attractive as a synthetic fuel due to its lightweight nature, abundance, and environmentally friendly combustion, producing only water vapor without any greenhouse gases. This potential makes hydrogen a viable candidate for reducing dependence on fossil fuels and controlling environmental pollution.

**FIGURE 2:**
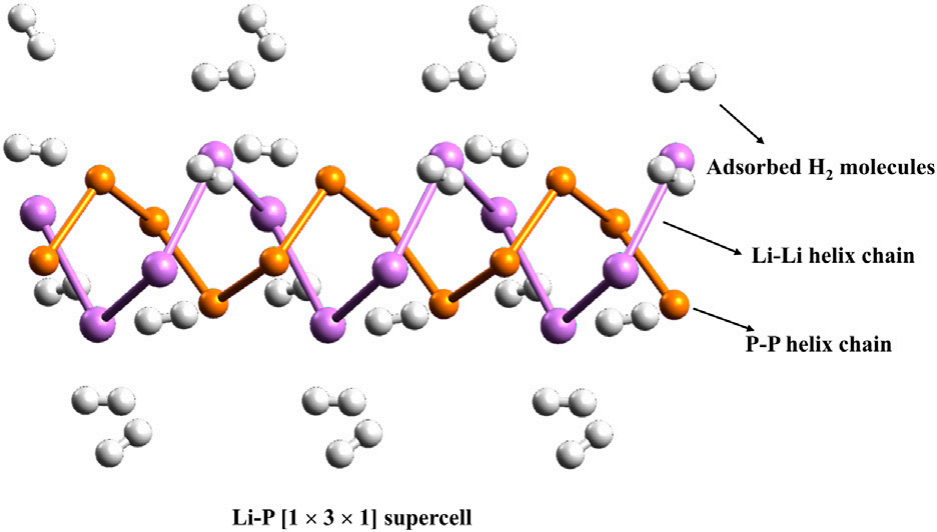
The 1 × 3 × 1 supercell of the infinite LiP chain.

In this context, we investigated the interactions between hydrogen (H_2_) molecules and the recently discovered double-helix structures of lithium phosphide clusters, Li_n_P_n_ (where n = 7–9), using DFT calculations. This computational study aims to understand the adsorption mechanisms and binding energies, offering insights into the potential of these unique structures for hydrogen storage applications. Binding energies per H_2_ molecule with these double-helices were found to range from 1.7 to 3.2 kcal/mol. Although these structures exhibit low polarizability, periodic DFT calculations with a 1 × 3 × 1 supercell configuration reveal that each lithium center within an infinite LiP chain can bind with two H_2_ molecules, achieving an average binding energy of 2.5 kcal/mol per H_2_ (see Figure 2). This corresponds to a substantial hydrogen uptake of 9.6 wt%, indicating strong potential for practical hydrogen storage.

**FIGURE 3:**
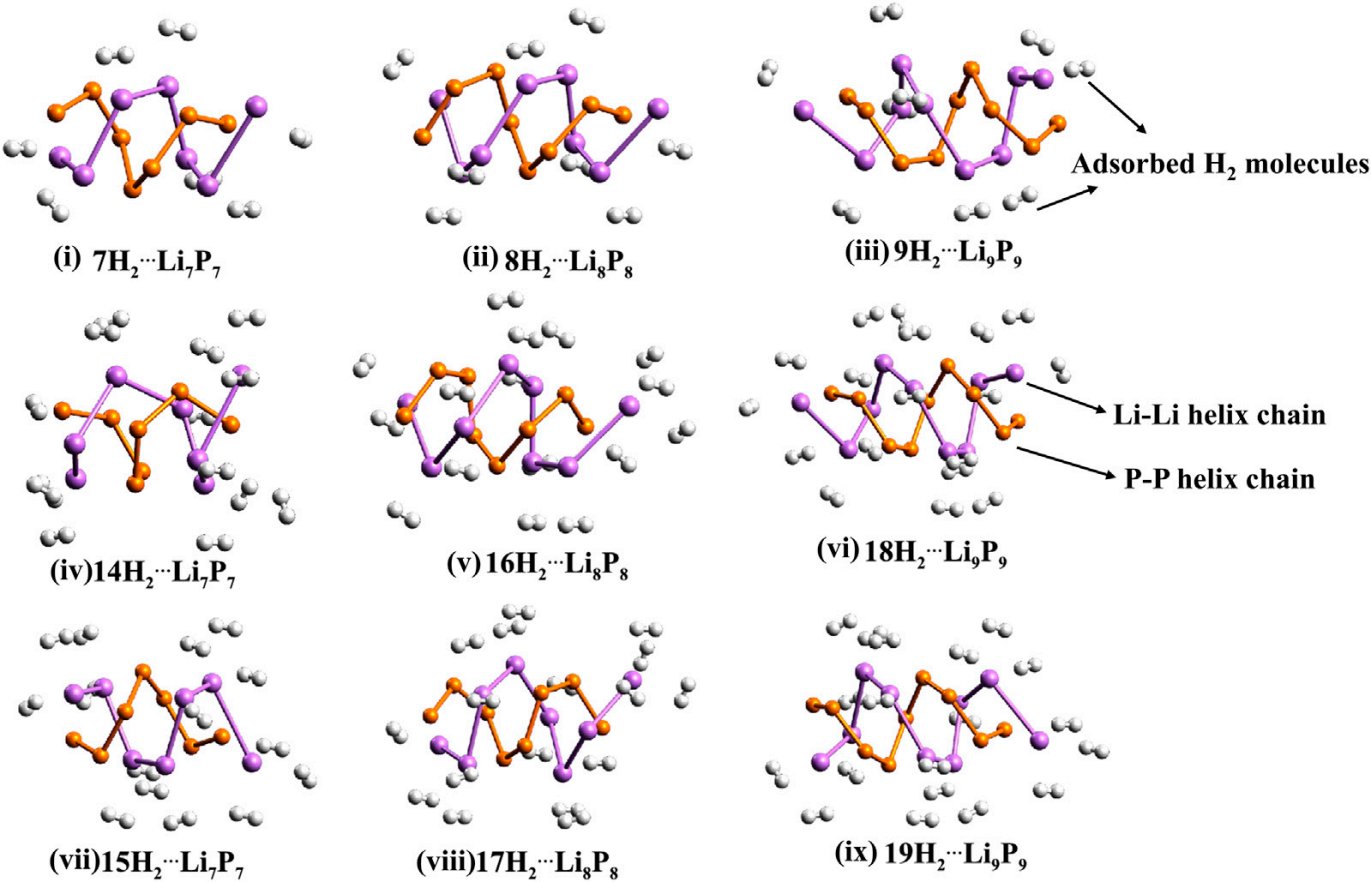
Inorganic double-helices made up of lithium and phosphorous (Li_*n*_P_*n*_; *n* = 7–9) with dihydrogen (H_2_) molecules.

The interaction between lithium centers and molecular hydrogen arises from both electrostatic and orbital contributions, as shown by EDA. Interestingly, a global minimum energy configuration search using a modified kick algorithm for H_2_@Li_7_P_7_ revealed that the most stable structure deviates significantly from the bare helical form, adopting a configuration that includes two P–H bonds. This finding suggests a preference for chemisorption under low-temperature conditions, as room temperature is not sufficient to achieve hydrogen chemisorption on LiP helices (see Figure 3).

Additionally, analysis of the electronic properties of the LiP helix structures indicates semiconducting behavior, characterized by a direct bandgap of 2.64 eV. This wide bandgap further underscores the potential utility of LiP helical structures in hydrogen storage applications, making them promising candidates for future energy systems focused on clean hydrogen storage and utilization.

Adding an extra H_2_ molecule to one of the terminal Li centers lowers the binding energy (*E*_b_) per H_2_ molecule to 1.7–1.8 kcal/mol. However, configurations where two H_2_ molecules bind per Li center, achieving 9.5 wt% gravimetric hydrogen storage, are more relevant for larger LiP helices. The decrease in *E*_b_ with increasing H_2_ loading is linked to the reduced positive charge on the Li centers. The H_2_···Li distances range from 2.004 to 2.536 Å, while H–H bond lengths remain largely unchanged or slightly elongated upon binding. These factors suggest that LiP helices are promising candidates for hydrogen storage.

Periodic unit studies of the LiP helix further support these findings, showing structural parameters consistent with the studied clusters. Minimal distortions upon H_2_ adsorption highlight the stability of LiP chains under high hydrogen density. Each Li center binds two H_2_ molecules with Li···H_2_ distances of 2.229–2.264 Å, and the *E*_b_ per H_2_ molecule is 2.5 kcal/mol, closely matching cluster results (see Table 2). The energy gap (Δ_H−L_) between the highest occupied molecular orbital (HOMO) and the lowest unoccupied molecular orbital (LUMO) is a simple indicator to check the stability of a given system. A species with high Δ_H-L_ value indicates that it is shy to accept or donate electrons and consequently it would show very poor reactivity and high stability. At the M06-2X/6-311 + G (d,p) level, the corresponding Δ_H-L_ values are found to be 3.81, 3.40 and 3.15 eV for Li_7_P_7_, Li_8_P_8_ and Li_9_P_9_, respectively. After the interaction with H_2_ molecules, it gets only slightly reduced, signifying that the presence of H_2_ does not alter the stability and reactivity of the systems considerably.

**TABLE 2:** The binding energy (E_b_, kcal/mol) per H_2_ molecule, natural charge at Li center (qLi, |e|) and weight percentage at the M06-2X/6-311+G (d,p) level.

Systems	Eb	qLi	wt%
Li_7_P_7_		0.40–0.75	
Li_8_P_8_		0.41–0.74	
Li_9_P_9_		0.41–0.74	
7H_2_ … Li_7_P_7_	3.2	0.20–0.61	5.01
8H_2_ … Li_8_P_8_	3.2	0.21–0.61	
9H_2_ … Li_9_P_9_	3.1	0.22–0.59	
14H_2_ … Li_7_P_7_	2.5	0.14–0.47	9.54
16H_2_ … Li_8_P_8_	2.5	0.17–0.29	
18H_2_ … Li_9_P_9_	2.5	0.18–0.36	
15H_2_ … Li_7_P_7_	1.8	0.14–0.37	10.01
17H_2_ … Li_8_P_8_	1.7	0.17–0.36	
19H_2_ … Li_9_P_9_	1.7	0.17–0.36	

We have analyzed the energy factors contributing to the H_2_···Li interaction using EDA, considering H_2_ molecules as one fragment and the LiP cluster as another. The results, summarized in Table 3, indicate that the interaction energy primarily arises from electrostatic and orbital contributions. Electrostatic interactions account for approximately 46%–47% of the total attractive forces, slightly surpassing the orbital contributions, which comprise about 42%–44%. In contrast, the dispersion interaction plays a relatively minor role, contributing only 10%–11% to the total attraction. This breakdown highlights the dominant roles of electrostatics and orbital interactions in stabilizing the H_2_···LiP system.

**TABLE 3:** EDA results of H_2_ bound Li_n_P_n_ clusters and 7Ng … Li_7_P_7_ at the PBE-D3/TZ2P//M06–2X/6–311+G (d,p) level and PBE-D3/TZ2P//M06–2X/def2-TZVP, respectively, taking H_2_ molecules or Ng atoms as one fragment and Li_n_P_n_ as another. All energy values reported here are per H_2_ or Ng and in kcal/mol. The values in parentheses are percentage contribution toward the total attraction, Δ*V*_elstat_ + Δ*E*_orb_ + Δ*E*_disp_.

Systems	Δ*E*_int_	Δ*E*_Pauli_	Δ*V*_elstat_	Δ*E*_orb_	Δ*E*_disp_
14H_2_ … Li_7_P_7_	−2.4	4.1	−3.0 (46.2)	−2.8 (43.6)	−0.7 (10.2)
16H_2_ … Li_8_P_8_	−2.3	4.1	−3.0 (47.0)	−2.7 (42.7)	−0.7 (10.3)
18H_2_ … Li_9_P_9_	−2.2	4.1	−2.9 (46.5)	−2.7 (42.4)	−0.7 (11.1)
7Ar … Li_7_P_7_	−1.8	2.4	−1.5 (35.0)	−1.6 (38.6)	−1.1 (26.3)
7Kr … Li_7_P_7_	−2.2	3.5	−2.2 (38.5)	−2.0 (34.4)	−1.6 (27.1)
7Xe … Li_7_P_7_	−2.6	4.9	−3.0 (39.6)	−2.6 (35.3)	−1.9 (25.1)
7Rn … Li_7_P_7_	−3.0	6.1	−3.9 (43.6)	−2.7 (30.2)	−2.4 (26.2)

#### 4.1.3 Hydrogen storage ability of functional graphyne nanotube

We systematically explored the hydrogen storage potential of a functionalized γ-graphyne nanotube (γ-GNT) modified with a small polar molecule, OLi_2_, attached to a boron-doped hexagonal boron nitride (h-BN) site using DFT calculations. The functionalization with OLi_2_ not only enhances the stability of the γ-GNT structure but also significantly improves hydrogen adsorption capacity compared to both bare and Li-adsorbed γ-GNT. Here, we discuss the stability, hydrogen binding energies, adsorption configurations, and the electronic properties of the functionalized γ-GNT, which make it a promising candidate for efficient hydrogen storage. The thermodynamic stability of the γ-GNT functionalized with OLi_2_ was confirmed through calculated binding energies. The results show that the structure of BOLi_2_, where OLi_2_ binds to a boron atom on the h-BN site of γ-GNT, is thermodynamically favorable. The stability of the BOLi_2_-γ-GNT configuration against the dissociation of OLi_2_ is evident from the negative binding energy values, which indicate that the adsorption of OLi_2_ is an exergonic process. This suggests that the interaction between OLi_2_ and the γ-GNT structure is energetically favorable, promoting the stability of the configuration and facilitating the hydrogen adsorption process.

This strong binding of OLi_2_ to the B atom ensures the retention of the functionalized structure under standard conditions, making it a reliable host material for hydrogen storage applications. The adsorption of Li atoms on γ-GNT and subsequent functionalization with OLi_2_ revealed significant differences in hydrogen storage capacity. In our study, Li atoms preferred to localize at the trigonal pole (TP) of the γ-GNT, while OLi_2_ showed a high binding affinity for the boron atom in a B, N-doped hexagonal ring connected to the acetylenic linkage. This site-specific adsorption of OLi₂ on B sites of γ-GNT results in a high-density polar surface, which enhances the material’s hydrogen adsorption capability through dipole-induced interactions. When hydrogen molecules were introduced to the system, OLi_2_-functionalized γ-GNT demonstrated a substantial improvement in hydrogen uptake over the bare and Li-adsorbed γ-GNT configurations. The presence of the polar OLi_2_ molecule allows each Li atom on OLi_2_ to adsorb up to three H_2_ molecules, while the negatively charged oxygen atom can adsorb an additional two H_2_ molecules. Consequently, each OLi_2_ group supports the adsorption of up to eight H_2_ molecules, significantly boosting the hydrogen storage capacity of γ-GNT. This uptake is particularly impressive given the non-dissociative adsorption mode of H_2_, allowing for rapid storage and release cycles. The binding energies of the adsorbed H_2_ molecules were found to fall within an optimal range (typically between 0.2 and 0.6 eV per H_2_ molecule), suitable for reversible hydrogen storage under practical operating conditions. This binding energy range is ideal as it strikes a balance between strong enough interactions to retain hydrogen molecules and weak enough interactions to allow desorption when needed. Additionally, the reaction enthalpies calculated for hydrogen adsorption further confirm the thermodynamic stability of hydrogen-loaded γ-GNT, highlighting the material’s ability to maintain structural integrity while storing significant quantities of hydrogen.

The stability and reactivity of the hydrogen-laden γ-GNT were also assessed through electronic structure analysis, specifically by examining the energy gap between the highest occupied molecular orbital (HOMO) and the lowest unoccupied molecular orbital (LUMO). The HOMO-LUMO gap of hydrogenated OLi_2_−functionalized γ-GNT remains sufficiently large to ensure stability, even under hydrogen loading. This indicates that the adsorption of H_2_ molecules does not significantly destabilize the electronic structure, supporting the material’s suitability for sustained hydrogen storage applications.

Natural Bond Orbital (NBO) analysis provided further insight into the charge distribution within the functionalized γ-GNT. The polar nature of the B-O and Li-O bonds in BOLi_2_-γ-GNT resulted in a positive charge on the Li atom and a negative charge on the O atom. This charge separation enhances the dipole moment of the functionalized site, strengthening the interaction with H_2_ molecules. The positively charged Li atom on each OLi_2_ group is capable of binding up to three H_2_ molecules through charge-induced polarization, while the negatively charged O atom can bind up to two H_2_ molecules, thus allowing eight H_2_ molecules to adsorb per OLi_2_ unit. This significant increase in hydrogen capacity highlights the potential of OLi_2_ functionalization as an effective strategy for improving hydrogen storage in γ-GNTs.

##### 4.1.3.1 Practical implications and advantages of OLi₂ functionalization

**FIGURE 4:**
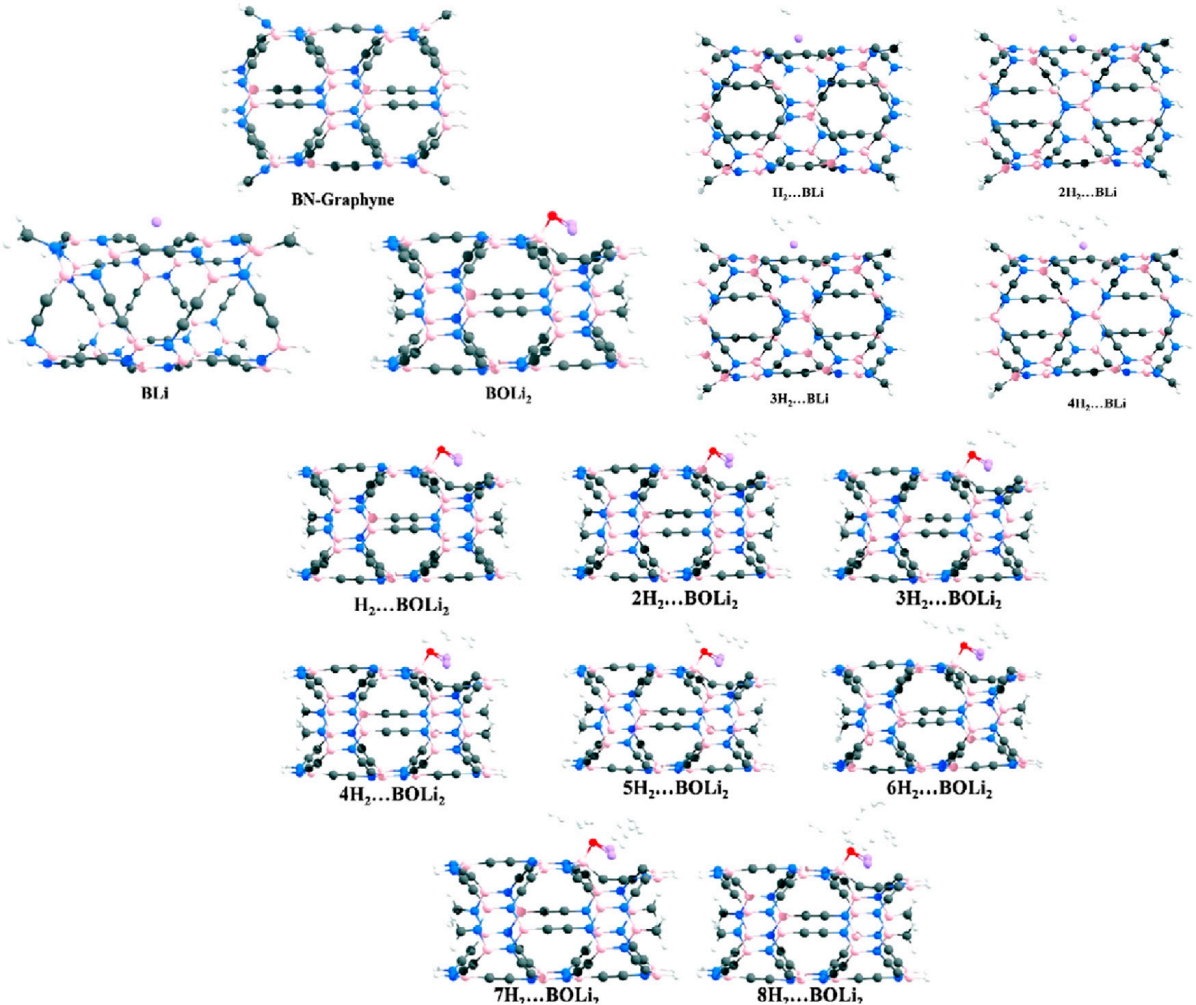
Structures of Geometries of optimized H_2_ … BLi and nH_2_ … BOLi_2_ at he ωB97X-D/6-31G (d, p) level where n = 1–8.

The functionalization of γ-GNT with OLi_2_ demonstrates several key advantages over unmodified γ-GNT and simple Li-adsorbed γ-GNT. The OLi_2_-functionalized γ-GNT not only stabilizes the structure thermodynamically but also introduces a strong dipolar field, which enhances hydrogen adsorption without requiring high pressures or temperatures. With a binding energy range that supports practical hydrogen storage and a high per-unit hydrogen uptake, OLi_2_−functionalized γ-GNT stands out as a promising candidate for hydrogen storage applications. The capability to store and release hydrogen efficiently under mild conditions could be particularly valuable for automotive and portable energy applications, where reversible hydrogen storage is crucial. Our DFT-based study demonstrates that OLi_2_−functionalized γ-GNT achieves a significantly enhanced hydrogen storage capacity (see Figure 4). By tuning the interaction energies through strategic functionalization, we achieve a balance between high capacity and stability. This approach provides a blueprint for developing advanced nanostructured materials for efficient hydrogen storage, marking a step forward in the search for viable hydrogen energy storage solutions.

#### 4.1.4 Hydrogen storage ability of metal acetylides and cyanides

The hydrogen storage capability of metal acetylides and cyanides has garnered significant attention due to their potential as efficient hydrogen adsorbents. These materials, when integrated with noble gases in their surrounding environment or within cryogenic matrices, exhibit enhanced stability and hydrogen binding characteristics. Noble gases such as argon and krypton, through weak van der Waals interactions, help stabilize the metal acetylide and cyanide complexes. This stabilization minimizes fluctuations in both the geometric and electronic structures of the metal centers, which is essential for maintaining favorable hydrogen binding sites. Quantum chemical studies have demonstrated that these cryogenic matrices play a critical role in stabilizing reactive intermediates and facilitating efficient hydrogen storage (Anderson et al., 2020; Ghosh et al., 2019). In this study, we explore the hydrogen storage capabilities of metal acetylide and metal cyanide compounds containing inserted Ng (Group 18) atoms, specifically with metals such as Cu, Ag, and Au, using the ωB97X-D/cc-pVTZ-PP computational method. Given the differing electronegativities and formal charges on the metal atoms within these insertion compounds, we expect variations in the interaction between the metal sites and hydrogen molecules.

**FIGURE 5:**
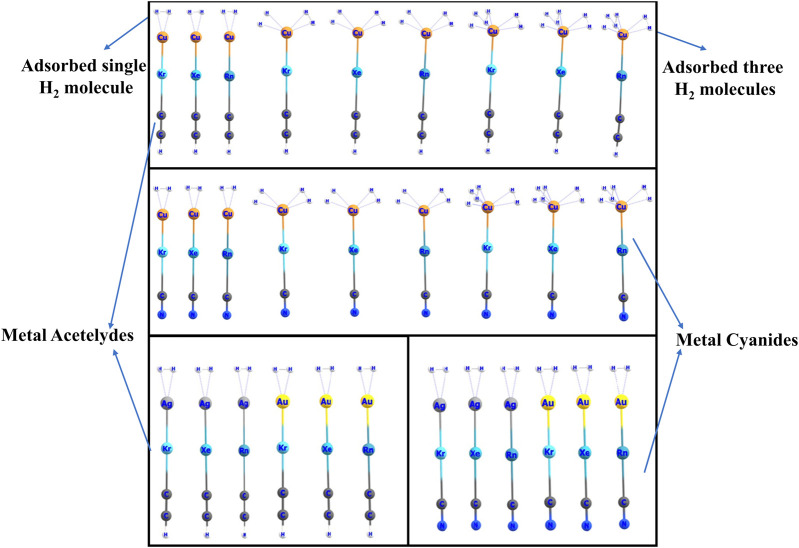
Optimized structures of H_2_ bound MNgCCH and MNgCN at the ωB97X-D/cc-pVTZ-PP level.

We calculate the adsorption energies, free energies of adsorption, and natural charges on atomic centers via natural population analysis, and perform energy decomposition analysis for systems of nH2···MNgCCH and nH2···MNgCN (where n = 1–3). The hydrogen adsorption capacities of both the strongest and weakest cases are also examined. It is found that both MNgCCH and MNgCN insertion compounds can adsorb up to three hydrogen molecules at the metal center (see Figure 5).

For the single-hydrogen adsorption configurations, the most stable structures exhibit a “T-shaped” geometry, while in the case of double-hydrogen adsorption, the stable configurations adopt a “Y-shaped” arrangement. For the trihydrogen systems, the geometry approximates a “Td-like” configuration. The negative values of Gibbs free energy changes suggest that the hydrogen adsorption process is thermodynamically spontaneous, indicating the potential viability of these compounds for efficient hydrogen storage applications.

This work sheds light on the promising hydrogen adsorption capabilities of MNgCCH and MNgCN insertion compounds and underscores their potential for use in hydrogen storage technologies.

## 5 Conclusion and future perspectives

Our study have provided crucial theoretical insights into hydrogen storage, offering qualitative predictions that drive material design. Through the ability to precisely calculate hydrogen binding energies, reaction processes, and mechanistic understanding, DFT has made it easier to optimize materials including carbon and nitrogen based nano-cages, noble metal based rare gas inserted acetylide and cyanides, and boron and nitrogen dopped graphyne frameworks. The development of high-performance hydrogen storage technologies that satisfy the needs of scalability, efficiency, and safety depends on these findings. By offering vital information on binding energies, adsorption processes, and chemical splitting behavior, the theoretical study improved our comprehension of hydrogen storage materials. Hydrogen storage materials have all seen experimental improvements as a result of DFT predictions on doping effects, catalytic mechanisms, and spillover processes. There are still practical difficulties, nevertheless, especially in obtaining high-capacity, reversible-release ambient temperature and pressure storage.
